# Photobiomodulation Therapy in Chronic Autoimmune Thyroiditis: A Systematic Review of Molecular Mechanisms and Clinical Applications

**DOI:** 10.3390/ijms27073007

**Published:** 2026-03-26

**Authors:** Venera Berisha-Muharremi, Alberta Humolli

**Affiliations:** 1 Faculty of Medicine, University of Prishtina, 10000 Prishtina, Kosovo; venera.berisha@uni-pr.edu; 2 Department of Endocrinology, University Clinical Centre of Kosovo, 10000 Prishtina, Kosovo; 3 Endomedica Polyclinic, Department of Endocrinology, 10000 Prishtina, Kosovo; 4 Faculty of Medicine, University of Ljubljana, 1000 Ljubljana, Slovenia

**Keywords:** photobiomodulation, low-level laser therapy, autoimmune thyroiditis, chronic autoimmune thyroiditis, thyroid laser therapy

## Abstract

Chronic autoimmune thyroiditis (CAT), a common autoimmune thyroid disorder, is the leading cause of hypothyroidism in iodine-sufficient regions and is characterized by thyroid autoimmunity, chronic inflammation, and progressive structural thyroid changes. Although levothyroxine (LT4) restores biochemical euthyroidism, it does not directly address the underlying autoimmune process, highlighting the need for adjunctive therapeutic strategies. Photobiomodulation (PBM), also known as low-level laser therapy (LLLT), has been proposed as a non-invasive intervention with potential immunomodulatory and tissue-level effects. A systematic narrative review was conducted following the Preferred Reporting Items for Systematic Reviews and Meta-Analyses (PRISMA) reporting principles. PubMed/MEDLINE, Google Scholar, and additional databases were searched for original human clinical studies evaluating PBM/LLLT in CAT, including studies using the term Hashimoto’s thyroiditis (HT), and reporting thyroid-related outcomes. Due to heterogeneity in study designs and PBM protocols, findings were synthesized narratively. Six eligible clinical studies published between 2010 and 2025 were identified. Across studies, PBM was associated with reductions in thyroid autoantibodies, improvements in thyroid hormone indices, and decreases in LT4 dose requirements. Longer follow-up studies reported ultrasonographic changes, while one sham-controlled trial demonstrated improvements in oxidative stress markers and quality of life (QoL) without short-term endocrine changes. However, current evidence is limited by the small number of human studies, heterogeneous PBM protocols, and the frequent use of concomitant interventions such as selenium or vitamin D. Overall, PBM may represent a promising adjunctive approach in CAT, although randomized sham-controlled trials are required before clinical implementation.

## 1. Introduction

CAT, also referred to in the literature as HT, is the most prevalent autoimmune endocrine disease and one of the primary causes of hypothyroidism in areas with sufficient iodine supply worldwide [1,2]. CAT is characterized by immune-mediated inflammation of the thyroid gland, lymphocytic infiltration, and the generation of thyroid-specific autoantibodies; subsequently, the structural and functional integrity of the thyroid tissue is progressively damaged [3,4,5]. Women are the predominant demographic affected by CAT, which tends to have a slow, chronic progression [6,7].

However, it is also important to note that hyperthyroidism is considered a form of autoimmune process in the body and is not due to a direct pathophysiological defect within the thyroid gland itself. The primary pathology in hyperthyroidism involves the immune system attacking the thyroid gland through autoimmune processes, with this being caused by an imbalance of the body’s immune response, along with the presence of genetic predispositions and exposure to certain external stimuli. Through these combined actions, there will be an ongoing destruction of the thyroid follicular cells.

The current standard of care for CAT involves hormone replacement therapy using LT4 to restore deficient levels of thyroid hormones [8]. Although it effectively restores biochemical euthyroidism, it does not specifically target the underlying immune response, chronic inflammation, oxidative stress, or structural abnormalities of the thyroid gland [4,9]. As a result, many patients will experience ongoing autoimmunity, abnormal thyroid morphology, or residual symptoms after their hormone replacement is optimized [4,9]. Therefore, there is a significant need for additional therapeutic approaches that focus on modifying both the pathophysiology of thyroid tissue and immune regulation versus simply replacing hormones.

PBM, also referred to as LLLT, is a non-invasive form of treatment where low-level laser or LED light, either red or near infrared, is used to modify cellular metabolism and/or tissue function without producing heat. PBM has been demonstrated to modulate mitochondria activity, oxidative stress balance, microcirculatory blood flow, inflammatory signaling pathways, and tissue repair mechanisms [10,11,12]. Given its mechanism of action, PBM has become increasingly studied as a potential therapeutic application for treating diseases involving inflammation and/or autoimmunity. Therefore, given the biological actions of PBM in diseases such as autoimmune thyroiditis, it provides a rational basis to study PBM as an adjunctive therapeutic strategy to treat localized inflammation and preserve some residual function of the thyroid tissue.

### Photobiomodulation: Cellular and Molecular Mechanisms Relevant to Chronic Autoimmune Thyroiditis

The main mechanism of action of PBM is at the cellular or sub-cellular levels, where light absorption by mitochondria (and therefore the entire cell) occurs due to the presence of photo acceptors (mitochondrial chromophores), especially cytochrome c oxidase (CCO), a critical component of the mitochondrial respiratory chain.

The activation of CCO leads to an increase in the rate of electron transport, increasing the membrane potential of the mitochondria and the amount of adenosine triphosphate (ATP) produced (i.e., improving the cell’s energy status), therefore supporting the repair processes of tissues and the survival of metabolically active cells (including thyroid follicular cells) [13,14].

In addition, PBM causes a temporary and controlled increase in reactive oxygen species (ROS) that acts as a secondary signaling mechanism rather than a source of cellular damage. This redox signal increases the expression of redox-sensitive transcription factors (such as nuclear factor erythroid 2-related factor 2 (Nrf2) and nuclear factor kappa B (NF-κB)), and, therefore, the endogenous antioxidant enzymes, such as superoxide dismutase and catalase, are activated.

Over time, these events will lead to a correction of the redox balance and a reduction in chronic oxidative stress, which is a hallmark of CAT since it involves the continuous generation of hydrogen peroxide, necessary for the production of thyroid hormones [9,10,11].

PBM has effects other than those related to mitochondrial and redox biology; specifically, PBM affects the signaling of immune response and inflammation. Both experimental and clinical studies have shown that PBM inhibits the expression of pro-inflammatory cytokines (such as tumor necrosis factor-α (TNF-α) and interferon-γ (IFN-γ)) and stimulates the production of anti-inflammatory mediators (such as transforming growth factor-β (TGF-β)).

This modulation of cytokines can potentially inhibit Th1-mediated immunity and increase the immune tolerance, thus preventing the autoimmune destruction of thyroid follicular cells and the production of thyroid autoantibodies [15,16].

Additionally, PBM modulates the nitric oxide (NO) signaling pathway and the microvascular function. The photonic-induced release of NO from CCO improves mitochondrial respiration, while stimulating the dilation of blood vessels and improving microcirculation.

In the thyroid gland, the increased perfusion may improve the viability of thyroid follicular cells, the synthesis of hormones, and tissue regeneration [17,18].

These mechanisms are especially relevant in CAT, where chronic autoimmune destruction of thyroid tissue is mediated by a combination of mitochondrial dysfunction, oxidative stress, and immune dysfunction.

**Figure 1:**
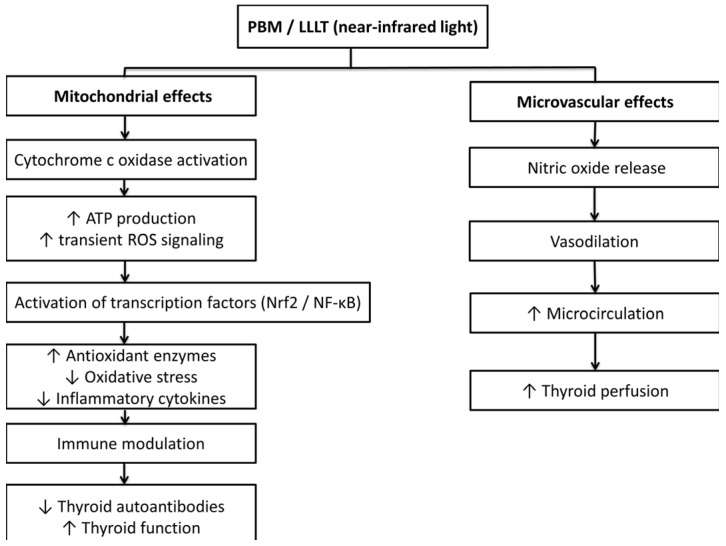
Proposed cellular and microvascular mechanisms of photobiomodulation (PBM) in chronic autoimmune thyroiditis. Arrows indicate the direction of change (increase ↑ or decrease ↓).

Collectively, these molecular and microvascular mechanisms provide biologic plausibility for the therapeutic effects reported in patients treated with PBM/LLLT for CAT (including improved thyroid hormone indices, reduced levels of thyroid autoantibodies, and reduced doses of LT4) [19] (Figure 1).

Therefore, the present review aims to evaluate the existing human clinical literature regarding PBM/LLLT for CAT, by evaluating the biochemical, structural, and self-reported patient outcomes and by relating them to the mechanisms of action described above.

Finally, we identify the most relevant gaps in the current evidence base and priorities for standardized sham-controlled trials with adequate follow-up.

## 2. Materials and Methods

A systematic review based on a narrative approach was conducted. The review adhered to PRISMA guidelines [20]. This systematic review was not prospectively registered in a public database (e.g., PROSPERO). The literature search was completed up to 31 December 2025. A detailed search strategy was used across PubMed/MEDLINE, Google Scholar, and other relevant databases to find studies that had been published up to the end date of the search before submitting the manuscript. Searches were performed using combinations of the following terms: “photobiomodulation”, “low-level laser therapy”, “Hashimoto thyroiditis”, “autoimmune thyroiditis”, “chronic autoimmune thyroiditis”, and “thyroid laser therapy”. An example PubMed/MEDLINE search string is as follows: (“photobiomodulation” OR “low-level laser therapy” OR LLLT) AND (“Hashimoto*” OR “autoimmune thyroiditis” OR “chronic autoimmune thyroiditis” OR “thyroiditis”).

Studies eligible for inclusion were original human clinical trials where participants were diagnosed with CAT, including studies using the term HT, and received PBM or LLLT. In order to be included, studies had to report a minimum of one clinically relevant measure, such as thyroid hormone levels, titers of thyroid autoantibodies, measurements of thyroid volume, or ultrasound findings or changes in LT4 dosage requirements. Excluded studies were those where all studies were conducted using animal or in vitro models, review articles or conference abstracts which did not contain complete data, or studies investigating non-autoimmune thyroid diseases.

**Figure 2:**
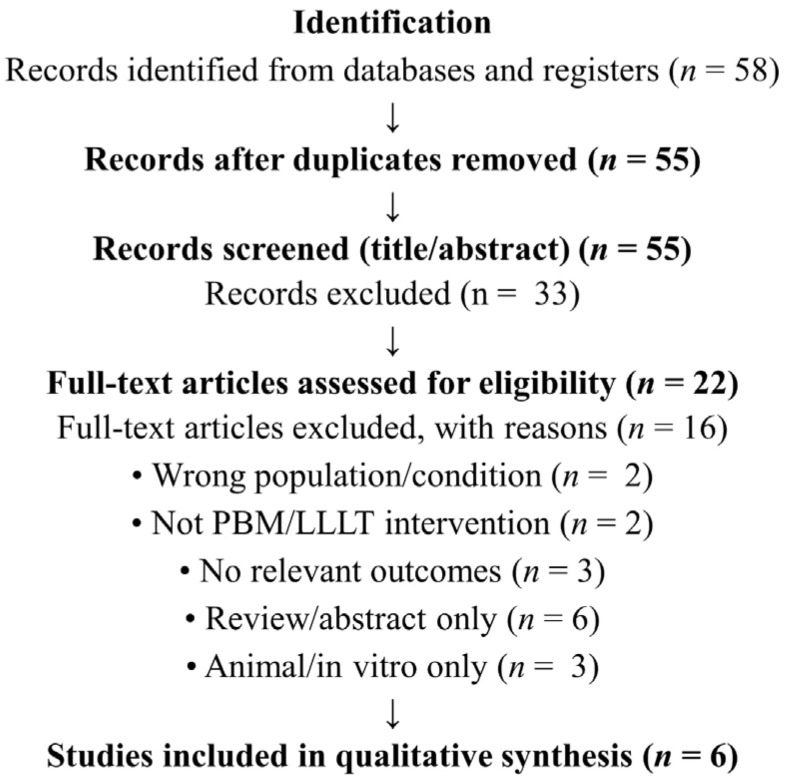
PRISMA flow diagram illustrating the study selection process for the systematic review of photobiomodulation in chronic autoimmune thyroiditis.

Two independent reviewers evaluated titles and abstracts to determine the relevance of each article and then further evaluated the full texts of potentially relevant studies. Duplicates were removed by hand prior to title and abstract screening. Screening and data abstraction were both done using Microsoft Excel. Any disagreements made during the selection process were resolved by consensus (Figure 2). Both data abstraction and evaluation of study quality were done independently. Study design, participant demographics, diagnostic criteria for CAT, PBM/LLLT parameters, co-intervention(s), and the results of the study were extracted from each study. Due to the high degree of heterogeneity among study designs, interventions, and outcome measures, a quantitative meta-analysis could not be done; therefore, results are synthesized narratively. Therefore, no formal risk-of-bias assessment was made; thus, results should be viewed with caution and with consideration given to the design of the study, control condition(s) (sham/placebo), and co-interventions. Additional methodological details are provided in the Supplementary Materials.

## 3. Results

### 3.1. Study Characteristics

A systematic review identified only a few human clinical studies that have evaluated the effects of PBM or LLLT in individuals with CAT. Six studies met the inclusion criteria for this qualitative review and are included in the synthesis. These studies were published during the years 2010–2025 and were conducted in Brazil, Turkey, and Kosovo [21,22,23,24,25,26].

These studies included a pilot open-label study, two randomized double-blind, single-blinded (placebo vs. active) and sham-controlled studies, a non-randomized cohort study, and two comparative studies. The sample size for these studies ranged from 15 to 350 participants. All of the studies recruited primarily females; the majority of the women had been on LT4 replacement therapy prior to enrollment [21,22,23,24,25,26].

All of the PBM protocols used near-infrared wavelengths ranging from 820 nm to 850 nm. The treatment durations ranged from short-term protocols lasting approximately three weeks to long-term follow-ups lasting up to twelve months. Many of the studies included nutritional supplements in addition to their PBM protocol. More than half of the studies included concomitant supplementation with selenium and/or vitamin D. The characteristics and interventions of all of the studies are summarized in Table 1.

The clinical outcomes reported by the studies that were included in this review were categorized into four areas: thyroid autoimmune disease, thyroid hormone parameters/LT4 dose, structural changes in the thyroid gland, oxidative stress, and QoL.

### 3.2. Thyroid Autoantibody Outcomes

Several studies have shown a decrease in the levels of thyroid autoantibodies. In a pilot clinical trial, the average levels of anti-thyroid peroxidase (anti-TPO) antibodies declined by approximately 41% (from 982 ± 232 IU/mL at baseline to 579 ± 272 IU/mL at the 9-month follow-up after PBM therapy) [21].

The non-randomized controlled cohort study showed that the PBM-treated group had a significantly larger decline in anti-TPO antibody levels compared to the group given supplementation alone (*p* = 0.0001) [23].

A second comparative study (parallel clinical trial) showed that there was a significant decline in anti-TPO levels in those who received PBM therapy with supplementation as opposed to those who only received supplementation (*p* < 0.05) [24].

Another comparative study conducted over 12 months found a statistically significant decline in both anti-TPO and anti-thyroglobulin (anti-TG) antibody levels in the PBM-treated group when compared to the control group (*p* < 0.05) [25].

### 3.3. Thyroid Hormone Parameters and Levothyroxine Dose

There was evidence of changes in both thyroid hormone outcomes and the amount of LT4 patients required across multiple studies.

In the pilot study, the average LT4 dose that patients received decreased from approximately 96 ± 22 µg/day before PBM treatment to 38 ± 23 µg/day at 9 months post-PBM treatment [21].

In the cohort study, the PBM-treated patients had a significantly greater increase in their triiodothyronine-to-thyroxine (T3/T4) ratio than control subjects (*p* = 0.0001). They also showed a significant decrease in their LT4 dose (*p* = 0.03) [23].

PBM combined with supplementation caused a significant increase in the T3/T4 ratio and a significant decrease in the amount of LT4 used when compared to supplementation alone (*p* < 0.05), in a parallel clinical trial [24].

Significant decreases in thyroid-stimulating hormone (TSH) levels and LT4 use were found in the PBM-treated patients compared to the controls at the end of a 12-month comparative study (*p* < 0.05) [25].

### 3.4. Structural Thyroid Outcomes

Structural changes in the thyroid, as measured by ultrasound, were identified in longer follow-up studies and were also found in studies where improvement in thyroid echogenicity occurred after PBM therapy, as was shown in the pilot clinical study [21].

PBM treatment was found to result in increased thyroid blood flow when compared to placebo in a randomized controlled clinical trial; specifically, there was an increase in inferior thyroid artery systolic peak velocity [22].

The comparative 12-month study demonstrated that combining PBM with supplementation resulted in a greater normalization of thyroid volume than supplementation alone (*p* < 0.05) [25].

### 3.5. Oxidative Stress and Quality of Life

While studies primarily examining endocrine and immunologic outcome measures have been conducted for the assessment of thyroid function, one randomized sham-controlled clinical trial has examined the effects of PBM on oxidative stress biomarkers as well as patient self-reporting QoL [26].

PBM therapy resulted in significant improvements in oxidative stress markers and patient-reported QoL compared with sham treatment (*p* < 0.05). However, no statistically significant short-term changes in thyroid hormone levels or thyroid autoantibody titers were observed during the 3-month follow-up period [26].

## 4. Discussion

### 4.1. Interpretation of Findings

The results of this systematic narrative review suggest that PBM has the potential to act as an adjunctive therapy in the treatment of CAT [21,22,23,24,25,26]. All of the clinical studies in the present review reported decreases in antibody levels against the thyroid (anti-TPO and/or anti-TG) and, similarly, many of the studies showed decreases in the dose required of exogenous LT4. These findings suggest a greater contribution of endogenously produced thyroid hormones compared to solely symptomatic control [21,23,24,25].

Selenium has an important function in thyroid physiology and redox balance by being incorporated into proteins containing selenium, like glutathione peroxidase and thioredoxin reductase. Selenium deficiency has been associated with higher oxidative stress and a more pronounced autoimmune response in individuals with CAT. Although many of the clinical trials reviewed contained patients who received selenium supplementation in conjunction with PBM therapy, it is difficult to determine the separate effect of PBM on the patient’s improvement due to the concurrent administration of these treatments.

In addition, vitamin D supplementation may affect both immune system regulation and thyroid autoimmunity and could be an additional variable that needs to be considered when determining if PBM provides a separate effect from other treatments.

In addition to the clinical findings, the studies using more contemporary study designs with defined dosimetry and longer follow-up times also reported changes in thyroid volume and other anthropometric parameters [25].

On the other hand, a single randomized sham-controlled trial assessing changes in oxidative stress and QoL reported improved redox biomarkers and patient-reported outcomes, but no corresponding short-term changes in thyroid hormone levels or autoantibody levels were noted [26].

This temporal dissociation suggests that PBM-induced biological effects may occur at the tissue or cell level prior to being evident in endocrine or immunologic markers.

Furthermore, the observed clinical findings of this review support previously established biologic effects of PBM including microcirculatory improvements, mitochondrial function enhancement, oxidative stress reduction, and immune system regulation [10,11,13,14,15,27].

Thyroid perfusion and redox equilibrium improvements may reduce inflammatory injury to follicular cells, thus preserving residual thyroid tissue functionality [9,17,18], which would explain the observed decreases in LT4 requirement and autoantibody titers in multiple studies, especially if PBM is administered early in the course of CAT with retained follicular reserves.

Additionally, the biologic effects of PBM are supported by a larger body of pre-clinical and experimental research. Pre-clinical studies have shown that PBM can affect mitochondrial function, down-regulate pro-inflammatory cytokines, modulate ROS and NO signaling, and improve microvascular perfusion and tissue repair. These biologic effects have been observed in a variety of inflammatory and autoimmune diseases, and they provide a mechanistic rationale for the clinical findings observed in patients with CAT [14,15,17,18,27].

However, due to differences in specific immune pathways of human autoimmune thyroid disease and the structure of tissues, there are limitations to the direct translation of data from experimental models to human autoimmune thyroid disease [14,15,17,18,27].

Due to the small sample size in many studies, variability in the dosing of PBM and definitions of outcome measures among the reviewed studies, as well as the fact that many of the reviewed studies employed co-interventions (selenium/vitamin D) [24,25], the strength of the evidence supporting the potential use of PBM as an adjunctive therapy in the treatment of CAT is limited.

### 4.2. Clinical Implications

The results of this study indicate that PBM could potentially be used in the future as a complementary treatment option for selected individuals with CAT. This could potentially include those with early or mild disease in which there is still thyroid tissue left and/or those on LT4 replacement therapy who continue to exhibit high levels of autoantibodies or experience ongoing symptoms even when their blood work indicates they are biochemically euthyroid.

It is important to note that, at present, PBM cannot be recommended as a standard practice for CAT due to the lack of consistent regulatory approval of PBM for CAT across regions and the lack of standardization of treatment parameters, including but not limited to wavelength, fluence, and treatment frequency. Therefore, until additional robust data is generated, PBM should be viewed as an investigative adjunct and used only within a monitored clinical environment under specialist supervision.

### 4.3. Standardization of Photobiomodulation Protocols

The literature on PBM is difficult to interpret due to a high degree of variability in PBM protocols. The majority of research reports have been using near-infrared light at wavelengths around 820–830 nanometers; however, there is significant variability in the other aspects of PBM protocol as well, such as power output, delivered fluence, number of treatment sites, and frequency of treatments. In addition, variations in the length of time that subjects were treated with PBM also limit comparisons among different studies. Furthermore, only a small percentage of studies have included control groups in their experimental design (i.e., “sham” controls), which limits the ability to determine whether observed effects are due to the actual use of PBM or a placebo effect. This is particularly important because light-based interventions may be susceptible to placebo effects, emphasizing the need for rigorously designed sham-controlled trials.

Another factor to consider when it comes to PBM is the dose–response relationship of PBM. PBM has been demonstrated to have a biphasic dose–response relationship (Arndt–Schulz Law) [13], where too little energy input does not result in a biological effect, but at an excessively high dose, energy can also be detrimental to cell function. Therefore, differences in wavelength, power density, fluence, and treatment frequency among different studies will likely greatly affect the outcome of therapy. The biphasic nature of the dose–response curve for PBM has been documented as a characteristic of this modality and emphasizes the need for careful optimization of the dose (dosimetry) to maximize the therapeutic benefit from PBM while minimizing the risk of either under-dosing with less than optimal levels of energy or over-dosing with energy that is greater than needed to produce a beneficial effect.

Additionally, many of these studies used supplemental micronutrients during the study (selenium and vitamin D), which may reflect the usual supportive care for patients with autoimmune thyroid disease but also make it difficult to attribute the results to the specific PBM treatment. Therefore, standardization of PBM protocols (e.g., dosimetry, anatomical treatment sites, treatment schedule) and validation of these standardized protocols will be essential for translating PBM into a clinical setting. These limitations in methodology mentioned above should also be considered when evaluating the safety of PBM.

### 4.4. Safety Considerations

Across the clinical trials listed above, there were no serious adverse events that could have been attributed to the PBM. No increases in thyroid nodularity or malignant transformation were noted in longer-term follow-up, including investigations conducted by Höfling et al. [21,22]. However, the available safety information is limited, and this is especially true for long-term exposure and for use in individuals with a history of thyroid nodules, prior neck radiation therapy, or increased risk of developing cancer (e.g., family history).

Although there is no reported increase in the occurrence of thyroid nodules and/or malignant transformation as a result of the current clinical trials, the number of participants in the clinical trials to date is relatively low, and the availability of long-term follow-up safety data is limited. Thus, it is advisable to exercise caution when using PBM on individuals who have known thyroid nodules and/or a history of thyroid malignancy; ideally, PBM would be avoided in this population until additional evidence regarding safety can be obtained.

Due to the close anatomic relationship of the thyroid gland to other important structures (carotid arteries, trachea, recurrent laryngeal nerve), it would appear prudent to carefully select patients who will receive PBM and have them monitored by specialists. It might also be wise to exclude patients who have a known or suspected thyroid malignancy and to perform regular imaging studies until additional safety information is available.

### 4.5. Limitations and Future Research Directions

Although preliminary results have been promising, the existing body of literature is limited due to a few key factors; there are only a handful of human clinical trials, as well as considerable variation among studies with respect to methodology (design), sample size, treatment protocols, and measurement of outcomes.

Short treatment protocols are unlikely to be sufficient for evaluating the therapeutic potential of PBM due to the chronic and slow-progressive nature of autoimmune thyroiditis. Longer-term follow-up is required to assess whether the biochemical or immunological changes seen post-PBM will translate into sustained clinical improvements. There are some long-term follow-up studies which suggest that there can be long-term benefits from PBM, with some studies showing that beneficial effects can last for years beyond the end of treatment [28]. These results would need to be confirmed in large randomized controlled studies.

Many of these studies use surrogate biochemical endpoints as opposed to outcomes that are centered around the patient, and in many cases, the follow-up time is not sufficient to assess whether patients can sustainably maintain the benefits they experienced from their participation.

Several methodological limitations should be taken into consideration when assessing the results of this review. The first is that few human clinical studies were identified for inclusion in the review. Additionally, many of these human studies had small sample sizes. Second, the studies examined in the review exhibited substantial heterogeneity regarding the parameters of PBM used to treat patients (i.e., wavelength, power output, fluence, frequency). Lastly, the use of various nutritional supplements in several of the studies reviewed (e.g., selenium and/or vitamin D) could represent an additional source of confounding variables, which could potentially affect both thyroid autoimmunity and oxidative stress independent of the effects of PBM.

Future investigations should include larger, multicenter, randomized, sham-controlled trials with longer-term follow-up, and more clinically relevant endpoints, such as symptom burden, QoL, ability to achieve and maintain reduced doses of LT4, and maintenance of normal thyroid function.

Additionally, mechanistic studies in humans, which could include assessment of thyroid perfusion imaging, oxidative stress markers, and immune profile, may provide additional insight into the biologic pathways through which any potential clinical effects are produced.

In order to better determine which patient populations would derive the greatest benefit from PBM, it may be beneficial to include more diverse patient groups within future studies, and to evaluate the effects of PBM across different stages of disease progression and residual volumes of thyroid tissue.

Cost-effective evaluations and standardized reporting of adverse events will be necessary for clinicians to make informed decisions regarding the application of PBM.

## 5. Conclusions

PBM could represent an interesting adjunctive option for the management of CAT, given its biological plausibility and the increasing clinical evidence supporting its application. The few available studies seem to show that PBM could be associated with a reduction in thyroid autoantibody levels, improvements in LT4 dose requirements, and in some cases, improvements in the structural parameters of the thyroid gland. However, at this time, the clinical evidence is still scarce and heterogeneous; therefore, it will be necessary to have well-designed, controlled (with a placebo or a sham), large-scale, randomized trials with a standardized protocol and a follow-up of sufficient duration before PBM can be used routinely in the clinical setting.

## Figures and Tables

**Table 1:** Summary of clinical studies investigating photobiomodulation in chronic autoimmune thyroiditis.

Study (Year)	Country	Study Design	Population	PBM Protocol	Reported Outcomes
Höfling et al. (2010) [21]	Brazil	Pilot open-label clinical study	15 patients with hypothyroidism due to CAT	830 nm, 50 mW, 10 sessions (2×/week for 5 weeks)	↓ LT4 dose; ↓ anti-TPO; improved thyroid echogenicity
Höfling et al. (2012) [22]	Brazil	Randomized, placebo-controlled clinical trial	43 patients with autoimmune hypothyroidism	830 nm, 50 mW, 10 sessions (2×/week for 5 weeks)	↑ Thyroid vascularization; ↑ inferior thyroid artery systolic peak velocity
Ercetin et al. (2020) [23]	Turkey	Non-randomized controlled cohort study	350 patients with HT (PBM + supplements vs. supplements only)	850 nm, 100 mW, 6 sessions over 3 days (8 points, 28.57 J/cm^2^ per point)	↑ T3/T4 ratio; ↓ anti-TPO; ↓ LT4 dose; improved QoL
Tunç et al. (2024) [26]	Turkey	Randomized sham-controlled clinical trial	46 patients with HT	PBM twice weekly for 3 weeks	↓ Oxidative stress markers (ROS, MDA, OSI); ↑ antioxidant enzymes (SOD, CAT); ↑ QoL; no short-term change in thyroid hormones or autoantibodies
Berisha-Muharremi et al. (2023) [24]	Kosovo	Parallel clinical trial	74 female patients with HT	820 nm, 200 mW, continuous mode, 20 s/point (8 points), 2×/week for 3 weeks, combined with selenium and vitamin D supplementation	↑ T3/T4 ratio; ↓ anti-TPO; ↓ LT4 dose
Berisha-Muharremi et al. (2025) [25]	Kosovo	Comparative clinical study with 12-month follow-up	98 female patients with HT	820 nm, 200 mW, continuous mode, 20 s/point (8 points), 2×/week for 3 weeks, combined with selenium and vitamin D supplementation	↓ Thyroid volume; ↓ anti-TPO, anti-TG, TSH; ↓ LT4 dose

## Data Availability

This study is based exclusively on data from previously published studies. No new data were generated or analyzed.

## Supplementary Materials

The following supporting information can be downloaded at https://www.mdpi.com/article/10.3390/ijms27073007/s1.

## Abbreviations

The following abbreviations are used in this manuscript:

CAT	Chronic Autoimmune Thyroiditis
HT	Hashimoto thyroiditis
PBM	Photobiomodulation
LLLT	Low-Level Laser Therapy
PRISMA	Preferred Reporting Items for Systematic Reviews and Meta-Analyses
QoL	Quality of Life
LT4	Levothyroxine
CCO	Cytochrome C Oxidase
ATP	Adenosine Triphosphate
ROS	Reactive Oxygen Species
Nrf2	Nuclear Factor Erythroid 2-Related Factor 2
NF-κB	Nuclear Factor Kappa B
TNF-α	Tumor Necrosis Factor-α
IFN-γ	Interferon-γ
TGF-β	Transforming Growth Factor-β
NO	Nitric Oxide
anti-TPO	Anti-Thyroid Peroxidase
anti-TG	Anti-Thyroglobulin
T3	Triiodothyronine
T4	Thyroxine
TSH	Thyroid-Stimulating Hormone
